# Correction of megavoltage cone‐beam CT images of the pelvic region based on phantom measurements for dose calculation purposes

**DOI:** 10.1120/jacmp.v10i1.2852

**Published:** 2009-01-27

**Authors:** Jean‐François Aubry, Joey Cheung, Olivier Morin, Alexander Gottschalk, Luc Beaulieu, Jean Pouliot

**Affiliations:** ^1^ Department of Radiation Oncology UCSF Helen Diller Family Comprehensive Cancer Center San Francisco CA U.S.A.; ^2^ Département de physique, génie physique et d'optique Université Laval Québec Canada

**Keywords:** megavoltage cone‐beam CT, cupping artifact, missing data artifact, dose calculation, pelvis imaging, prostate cancer

## Abstract

Megavoltage cone‐beam CT (MVCBCT) is an imaging technology that provides a 3D representation of the patient in treatment position. Because it is a form of x‐ray tomography, MVCBCT images give information about the attenuation coefficients of the imaged tissues, and thus could be used for dose calculation. However, the cupping and missing data artifacts seen on MVCBCT images can cause inaccuracies in dose calculations. To eliminate these inaccuracies, a correction method specific to pelvis imaging and based on phantom measurements has been devised. Pelvis‐shaped water phantoms of three different sizes were designed and imaged with MVCBCT. Three sets of correction factors were created from the artifacts observed in these MVCBCT images by dividing the measured CT number by the predefined CT number for water. Linear interpolation is performed between the sets of correction factors to take into account the varying size of different patients. To compensate for the missing anatomy due to the limited field of view of the MVCBCT system, the MVCBCT image is complemented with the kilovoltage CT (kVCT) image acquired for treatment planning. When the correction method is applied to an anthropomorphic pelvis phantom, the standard deviation between dose calculations performed with kVCT and MVCBCT images is 0.6%, with 98% of the dose points agreeing within ±3%. With uncorrected MVCBCT images this percentage falls to 75%. An example of dose calculation performed with a corrected clinical MVCBCT image of a prostate cancer patient shows that changes in anatomy of normal tissues result in variation of the dose distribution received by these tissues. This correction method enables MVCBCT images to be used for the verification of the daily dose distribution for patients treated in the pelvis region.

PACS numbers: 87.57.Q‐ Computed tomography, 87.57.cp Artifacts and distortion

## I. INTRODUCTION

The goal of radiation therapy is to deliver a prescribed dose to cancerous cells while minimizing the adverse effects to nearby normal tissues. During the past twenty‐five years, advancements in diagnostic and 3D imaging like kilovoltage computed tomography (kVCT)^(1)^ and magnetic resonance imaging (MRI),^(2)^ and in treatment techniques such as 3D conformal therapy^(3)^ and intensity‐modulated radiation therapy^(4)^ (IMRT), have all contributed to bring practice closer to the ideal treatment. New technologies allowing in‐room imaging have recently brought increased interest in treatment verification strategies like image‐guided radiotherapy^(5)^ (IGRT). These technologies, which include kilovoltage cone‐beam CT^(6)^ ^,^ ^(7)^ (kVCBCT), megavoltage CT^(8)^ (MVCT) and megavoltage cone‐beam CT^(9)^ ^,^ ^(10)^ (MVCBCT), are also considered to push treatment verification from positioning and localization to delivered dose distribution assessments.^(11)^ ^,^ ^(12)^ In current practice, only the dose that is intended to be delivered is known with certainty. The ability to perform dose calculations with images acquired moments before treatment delivery would provide important information about the dose that is actually received by the patient. This would allow the development of treatment plan adaptation schemes^(13)^ ^,^ ^(14)^ and could improve the biological models used to predict treatment outcomes.^(15)^

Dose calculation with MVCBCT images has already been suggested as an effective way to recalculate the “dose of the day”.^(14)^ ^,^ ^(16)^ However it has been noted that dose calculation using cone‐beam CT imaging techniques could be impaired by cupping and, in some cases, missing data artifacts.^(17)^ ^,^ ^(18)^ ^,^ ^(19)^ ^,^ ^(20)^ Various methods to solve these problems have recently been suggested. Some of these techniques use Monte Carlo simulations,^(18)^ while others use a reference image coming from a different imaging system to eliminate most of the artifacts.^(20)^ ^,^ ^(21)^ Another approach consists of characterizing the artifacts with calibration phantoms in order to remove them on patient images. Morin et al.^(17)^ have recently proposed such a method to correct MVCBCT images of the head and neck region. The purpose of the present work is to rework and extend this concept to pelvis MVCBCT imaging.

The structure of this paper is as follows. An overview of the problem of the cupping and missing data artifacts in pelvis MVCBCT images is given in section II.A. The method proposed to correct for these artifacts is then described in detail in section II.B. The correction method was applied to anthropomorphic phantom images and to prostate cancer patient images, as explained in sections II.C and II.D, respectively. The results of dose calculations using these images are presented in section III and discussed in section IV.

## II. MATERIALS AND METHODS

### A. Pelvic imaging with megavoltage cone‐beam CT

Megavoltage cone‐beam CT is a technology that allows in‐room 3D imaging of the patient in treatment position using a conventional linear accelerator. At the UCSF Helen Diller Family Comprehensive Cancer Center the MVision MVCBCT system is installed on two Oncor accelerators and one Primus (Siemens OCS, Concord, CA). The x‐ray imaging source is a 6‐MV treatment beam, and the detector consists of an AG9 (PerkinElmer Optoelectronics, Fremont, CA) amorphous silicon flat panel mounted on the gantry. The source detector distance is set to 145 cm. Transmission images are recorded over an arc rotation of 200°, and a cylindrical volume of 27.4 cm diameter and 27.4 cm length is reconstructed using a modified Feldkamp algorithm. More details about the system can be found in the literature.^(9)^ ^,^ ^(14)^ ^,^ ^(22)^ Before imaging, the patient is positioned on the treatment table using skin marks that correspond to the treatment isocenter. This implies that for the cone‐beam imaging the patient will be aligned within roughly 1 cm of the planned patient position. The resulting MVCBCT image consists of 1 mm cubic voxels, where voxel values have a linear relationship to the attenuation coefficient of the tissue.

Because of this property of x‐ray imaging, MVCBCT images can be used for dose calculation with proper CT number calibration. However, such images exhibit a cupping artifact, characterized by a darker region in the middle of the image. The cupping artifact increases with patient size because it is caused by a mixture of scattered photons reaching the detector and off‐axis softening of the beam. Moreover, in pelvic imaging, a missing data artifact caused by an imaging volume smaller than a normal sized adult patient is also present. The effect of this artifact is to increase the CT number in the outer ring of the axial cross sections. This imaging artifact also increases with object size. The cupping and missing data artifacts combine to create substantial non‐uniformity in the image, especially for pelvic imaging. This can be observed in Figure 1a, which shows lateral and longitudinal profiles taken from pelvis‐shaped water phantoms MVCBCT images of three different sizes. With a perfect imaging system, these profiles would be flat. To correct for this non‐uniformity, a simple method involving calibration phantoms and correction factors has been developed.

**Figure 1:**
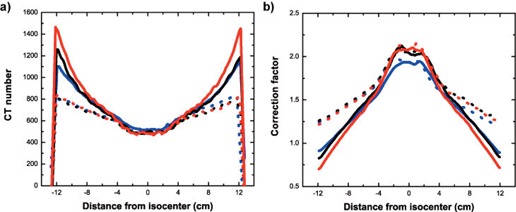
CT numbers and correction factors profiles. (a) Profiles of CT numbers from MVCBCT images of pelvis water phantoms of different sizes. (b) Profiles of correction factors obtained from the same three water phantoms; blue: phantom A (small size); black: phantom B (medium size); red: phantom C (big size). Solid lines: lateral profiles. Dashed lines: longitudinal profiles.

### B. Calibration phantoms and correction factors

Three calibration phantoms were designed to quantify the non‐uniformity of pelvic MVCBCT images as a function of imaged object size. They were custom‐made out of abutted plastic containers, containing a plastic watertight contractor bag which was filled with water (Figure 2). Their shape represented roughly a human pelvis, with average thicknesses of 25.4 cm (phantom A), 29.8 cm (phantom B), and 39.0 cm (phantom C) when full. Their average thickness as a function of imaging angle is plotted in the black curves of Figure 3. Even though they were of different sizes, all phantoms were thicker in the lateral direction than in the anteroposterior (AP) axis. They were also designed to fully occupy the inferior‐ superior dimension of the imaging system.

**Figure 2:**
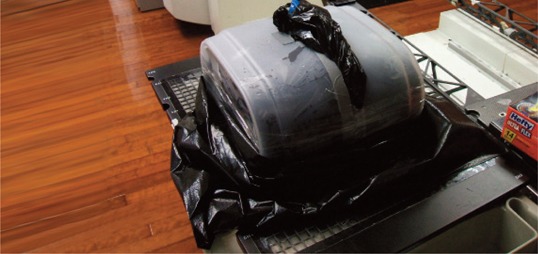
Example of a custom‐made pelvic water phantom. This picture shows phantom B.

**Figure 3:**
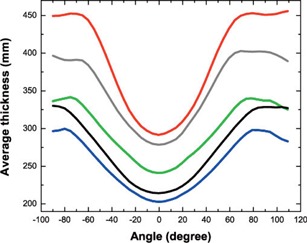
Average thickness of four phantoms and one patient as a function of MVCBCT acquisition angle; blue: phantom A; green: phantom B; red: phantom C; grey: anthropomorphic phantom used for validation; black: prostate cancer patient.

The phantoms were imaged using the same protocol used for patients but with a total x‐ray exposure of 20 monitor units (MU) to reduce the noise. The reconstructed 3D images were filtered with a cubic averaging kernel of seven voxels wide to reduce the noise. Correction factors were created based on each of these images using Equation 1, so that each voxel k of the water phantom images led to a corresponding correction factor CF(k). At our institution, MVCBCT images are usually calibrated so that water has a CT number of 1000; this was the value used for ${\text{CT}}_{\text{Water}}$.

(1) $$CF(k)=CT_{Water}/I(k)$$

Three sets of correction factors (sets A, B and C) were created from the phantoms. To correct a MVCBCT image, a simple multiplication of this image by a set of correction factors is performed on all voxels.

In practice, patients come in all sizes and it is very unlikely that they will match exactly with one of the calibration phantoms. To accommodate this, interpolation is carried out between the sets of correction factors. This process requires knowledge of the thicknesses of the calibration phantoms and the size of the particular patient in question. Several methods could be used to obtain this information. Since all of our patients treated in radiation oncology are scanned with a kVCT for treatment planning purposes, these images were used in this study to determine the average thickness over the range of MVCBCT acquisition angles. A thickness map for angles ranging from −90° to 110° is created at 5° increments using a radiological path algorithm.^(23)^ The average thickness for each map is computed creating curves similar to the ones shown in Figure 3. The differences of these thicknesses relative to the calibration phantom thicknesses are then computed and averaged over all angles, thus suggesting what proportion of each of the correction factors sets to use. A maximum of only two sets are considered for a given patient. The same interpolation is applied equally to all voxels of the two sets considered.

### C. Validation on anthropomorphic phantom

The accuracy of the correction method was validated using a Rando anthropomorphic phantom of the pelvis region. Because this phantom is very small when compared to the size of the average patient at our institution, it was placed in a bigger custom‐made water phantom. This phantom was built by placing the Rando phantom inside phantom B, and then adding bolus sheets and wax on the sides, and also adding a slab of solid water underneath it. The resulting thickness of this phantom is shown in Figure 3, with an overall average thickness of 35.1 cm.

The phantom was imaged both with a kVCT and MVCBCT system. For the kVCT imaging, a standard pelvis acquisition protocol was used, while the exposure for the MVCBCT image was 20 MU. The kVCT image was used to obtain the thickness of the phantom, and the corresponding set of correction factors was determined. In this case the interpolated correction factors were created using 40% of set B and 60% of set C. These correction factors were then applied to the MVCBCT image. This corrected MVCBCT image was then completed for its missing part with the kVCT image, creating an image labeled “corrected MVCBCT+”. This completion step consists of registering the kVCT with the MVCBCT image, and replacing the region where there is an overlap in the kVCT image with the MVCBCT image. This procedure has already been described in more detail in a previous publication.^(21)^ An uncorrected MVCBCT+ image was also created. Cross‐sections of the kVCT, uncorrected MVCBCT, uncorrected MVCBCT+ and corrected MVCBCT+ images are shown in Figure 4.

**Figure 4:**
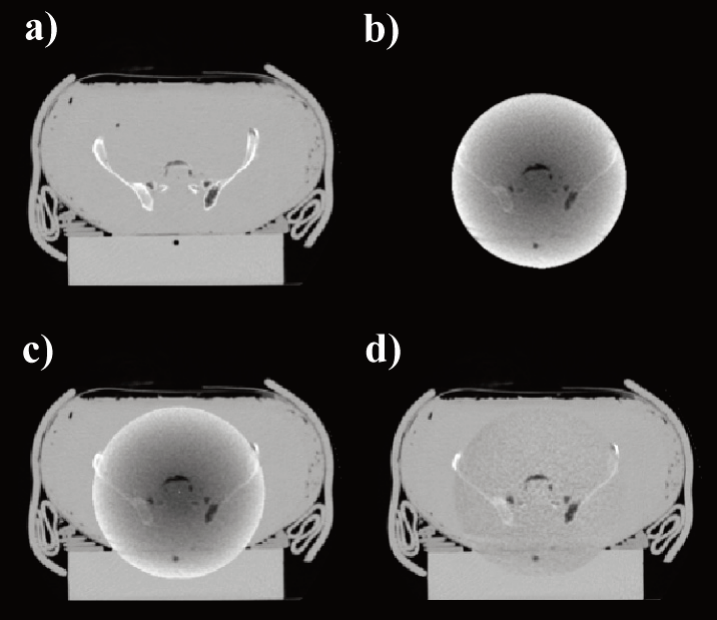
Cross sections of kVCT and MVCBCT images of an anthropomorphic pelvic phantom: (a) the kVCT image; (b) the uncorrected MVCBCT image; (c) the uncorrected MVCBCT+ image; and (d) the corrected MVCBCT+ image for the anthropomorphic phantom.

An IMRT treatment plan copied from an actual prostate cancer patient and consisting of seven beams was applied to the kVCT, uncorrected MVCBCT+ and corrected MVCBCT+ images.

Percent dose differences (PDDiffs) between the kVCT dose distribution and those obtained with the MVCBCT+ images were computed using Equation 2.

(2) $$PDDiff=\frac{\left(D_{CB+}-D_{kVCT}\right)}{D_{kVCT}}\times 100$$

With the PDDiffs plotted in a histogram, the parameters of a Gaussian distribution (Equation 3) were fitted to the measured distribution. The fitting procedure was performed with a non‐linear least‐square Levenberg‐Marquardt algorithm.

(3) $$y=A\times \text{exp}\left(\frac{-{\left(x-x_{c}\right)}^{2}}{2\sigma^{2}}\right)$$

### D. Patient case

An MVCBCT image of a patient treated for prostate cancer was acquired with an exposure of 10 MU during the course of his treatment. The MVCBCT projections were processed using a diffusion filter^(24)^ prior to reconstruction to increase the contrast to noise ratio.^(22)^ This pre‐reconstruction filtering increases the contrast‐to‐noise ratio in the reconstructed image by a factor equivalent to at least a five‐fold increase in exposure. The average thickness of this patient as a function of projection angle is shown in Figure 3. The overall average thickness was 27.5 cm. The MVCBCT image was corrected using the method explained in section IIB. The treatment plan was delivered with a step and shoot IMRT technique using 7 beams and 45 segments.

The bladder, rectum and prostate were contoured using the MVCBCT image by the radiation oncologist (Alexander Gottschalk) who had originally contoured these structures on the planning kVCT Dose calculation was then performed on the MVCBCT image, using the same beams as the treatment plan, with the beam isocenter located exactly where it was during the actual treatment delivery. Dose‐volume histograms (DVHs) were computed for all three structures and compared with the treatment plan DVHs.

## III. RESULTS

### A. Correction factors

Lateral and longitudinal profiles of the three sets of correction factors are shown in Figure 1b. Their magnitude runs from roughly 0.7 in the periphery to 2.2 on axis. The differences between successive correction factors sets do not exceed about 0.2 for a given voxel. Their shape is inverted from the profiles of the calibration images as a result of Equation 1.

### B. Validation on anthropomorphic phantom

Figure 5 compares histograms of the PDDiffs obtained with the uncorrected and corrected MVCBCT+ images. Without correction, errors in the dose calculation vary between −4% and 8%, with only 75% of the dose distribution being within ±3%. With the correction applied to the MVCBCT image, the errors are reduced to a range of −3% to 3% in 98% of the dose distribution. The fitted standard deviation σ for the Gaussian distribution was 0.6% with a 95% confidence interval ranging from 0.5% to 0.7%. Figure 6 illustrates the distribution of the errors obtained with both images along with the Gaussian fit.

**Figure 5:**
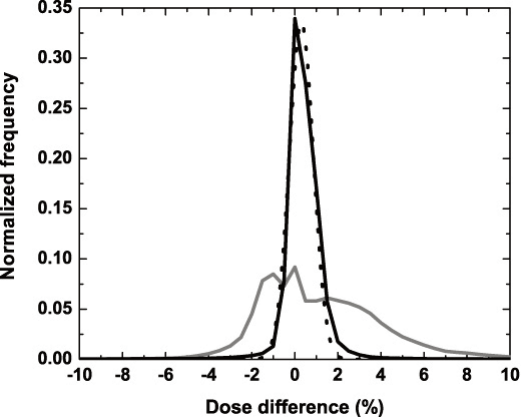
Percent dose difference histograms. The PDDiffs between the dose distributions obtained with kVCT and the uncorrected CB images are in solid gray, and the kVCT and the corrected MVCBCT+ images are in solid black. The fitted Gaussian function is shown by the black dashed line.

**Figure 6:**
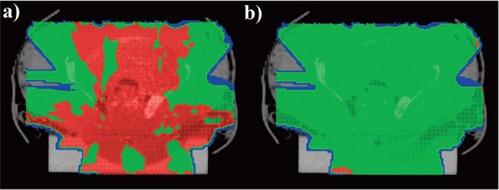
Cross section of the percent dose differences. was obtained with the uncorrected MVCBCT+ image of the anthropomorphic phantom; and 6b was obtained with the corrected MVCBCT+ image of the anthropomorphic phantom. Red shows a PDDiff of 3% or more; green shows a PDDiff of ±3%; and blue shows a PDDiff of −3% or less.

### C. Patient case

Interpolation of this patient's average radiological thickness lead to correction factors that used 56% of set A and 44% of set B. Figure 7 shows the contours from both the kVCT and MVCBCT images, along with the corresponding DVHs, for the bladder, rectum and prostate. Since very limited anatomical changes occurred for the prostate, the DVHs show only slight differences between the planning and treatment images. However, the bladder was larger on the MVCBCT image than it was on the planning kVCT image. This difference shows up on the DVHs, where the bladder receives more dose during treatment than that planned. Small variations in rectum contours and filling also lead to differences in the low dose region of the DVHs.

**Figure 7:**
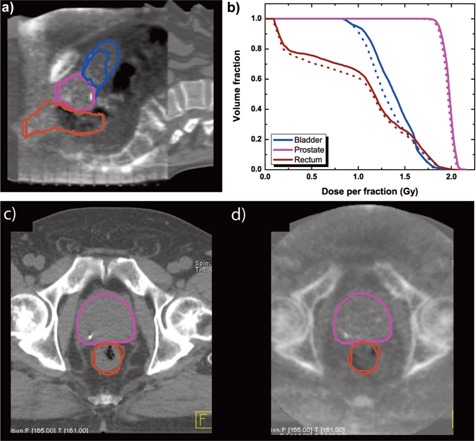
Dose calculation with clinical kVCT and MVCBCT images. (a) Sagittal slice showing the overlaid contours of the prostate (pink), rectum (brown) and bladder (blue) from the planning kVCT and the MVCBCT image. The bigger bladder contour is from the MVCBCT image. (b) Dose‐volume histograms of these structures for treatment planning (dotted lines) and obtained with the MVCBCT image (solid lines). (c) Close up of the prostate and rectum contours on the planning CT image. (d) Close up of the prostate and rectum contours on the MVCBCT image.

## IV. DISCUSSION

Three calibration phantoms of different size were designed to obtain correction factors. The phantom sizes were chosen so that they cover the majority of patient sizes observed clinically. Characterizing the cupping and missing data artifacts for more than one phantom size is important because different sizes lead to different correction factors. As seen in Figure 1.b, phantoms A and B lead to similar off‐axis correction factors but different on‐axis correction factors. Moreover, phantoms B and C lead to similar on‐axis correction factors but different off‐axis ones; therefore all three phantoms are needed. Even though these differences seem small on Figure 1.b, a 0.1 error in a correction factor that has a value around 1 is still 10%, which is an error important enough to cause unacceptable dose calculation errors. For example, if the MVCBCT image of phantom C is corrected with the correction factors obtained from phantom B, only 68% of the dose calculation is accurate within ±3%. Because interpolation between three sets of correction factors is sufficient to provide dose calculation with a discrepancy of under 1%, adding extra phantoms with other thicknesses would not meaningfully improve the correction method.

The calibration phantoms were positioned with their geometric center roughly at the imaging isocenter for the acquisition of the MVCBCT images. Corrected MVCBCT+ images acquired with offsets of up to 3 cm lead to dose calculations that matched the reference within ±3% for 97% of the volume. Because the treatment isocenter is usually placed near the geometric center of the patient (along the lateral and AP axes) during treatment planning, the exact positioning of the patient is not an issue for this correction method.

A possible limitation of this method is the use of the kVCT image to complement the missing parts of the MVCBCT image. However, by registering the two images based on bony landmarks before the creation of the MVCBCT+, a structural continuity is established between both parts of the MVCBCT+ image. If the bony structures match well between the two images, the it is a fair assumption that the overall shape of the patient did not change by much. When acquiring in‐room images of prostate cancer patients, for example, it is more the variations of internal structures such as prostate, rectum and bladder that are of interest. This technique has been used with a commercial MVCT imaging system before.^(25)^ To avoid resorting to another image to complement the MVCBCT image, one solution consists of allowing the flat panel detector to move laterally. Therefore a 360° acquisition with the detector laterally offset provides a wider field of view.^(26)^ The system that was used for this study does not provide such functionality.

Currently the average thickness of the patient along different projection angles is obtained with the kVCT acquired for treatment planning. However there exists other means to obtain that information. Images from other modalities, such as MRI, could be used. In this case, assuming that the whole body contains water would be a sufficient approximation of thickness. There is also a way to obtain the patient's thickness without any imaging other than the MVCBCT image, as described recently by Maltz et al.^(27)^ In this method, only the data from the truncated CT image is needed to reconstruct a homogeneous, elliptical approximation of the patient's shape and thickness.

The correction method presented in this paper is based on phantom measurements, an idea that was already explored in the work of Morin et al.^(17)^ However, their method was based on an 3D fitting of the cupping artifact to an ellipsoid function and did not involve interpolation between phantom sizes to account for the varying degrees of cupping artifact as a function of size. The new method presented here is the only one, to the best knowledge of the authors, that can correct for both cupping and missing data artifacts in MVCBCT images of the pelvis. Another type of correction method that has been proposed aims at correcting the cupping artifact at the projections level, before image reconstruction. These methods can be based on Monte Carlo simulations^(18)^ or on experimental models.^(28)^ Although they also report very good results, none of these methods have been used to correct missing data artifacts. Therefore these methods have only been applied clinically to head‐and‐neck images.

Dose calculation on an actual patient MVCBCT image showed that anatomical changes that lead to dose discrepancies between the treatment plan and what actually happens during treatment can be detected. However, if no anatomical changes occur, the planning and treatment DVHs are similar, within the 0.6% standard deviation of dose calculations between MVCBCT and kVCT images.

## V. CONCLUSIONS

A simple cupping and missing data artifacts correction method was created for pelvis MVCBCT images. The method is based on inexpensive custom‐made calibration phantoms and could be applied or repeated in any other treatment center equipped with an MVCBCT system. Dose calculations with corrected MVCBCT images agree with those obtained with kVCT images within ±3% for 98% of the volume, while the standard deviation of the differences is 0.6%. This correction method can be applied to patient images and allows for the verification of delivered dose during the course of a treatment.

## ACKNOWLEDGMENTS

This work was partly funded by Siemens Oncology Care Systems. One of the authors, Jean‐François Aubry, wishes to acknowledge financial support from the Natural Sciences and Engineering Research Council of Canada (NSERC).

## References

[1] Ling CC, Rogers CC, Morton RJ, editors. Computed Tomography in Radiation Therapy. New York (NY): Raven Press; 1983 284

[2] TenHaken RK , Thornton AF Jr , Sandler HM , et al. A quantitative assessment of the addition of MRI to CT‐based, 3‐D treatment planning of brain tumors. Radiother Oncol. 1992;25:121–131. 1332134 10.1016/0167-8140(92)90018-p

[3] Fraass BA . The development of conformal radiation therapy. Med Phys. 1995 Nov;22(11 Pt2):1911–1921. 8587545 10.1118/1.597446

[4] Webb S . Intensity‐modulated radiation therapy. 1st ed. Bristol (UK): Institute of Physics Publishing; 2001 435

[5] Ma CM , Paskalev K . In‐room CT techniques for image‐guided radiation therapy. Med Dosim. 2006 Spring;31(1):30–39. 16551527 10.1016/j.meddos.2005.12.010

[6] Jaffray DA , Siewerdsen JH , Wong JW , et al. Flat‐panel cone‐beam computed tomography for image‐guided radiation therapy. Int J Rad Oncol Biol Phys. 2002;53(5):1337–49. 10.1016/s0360-3016(02)02884-5 12128137

[7] Oelfke U , Tucking T , Nill S , et al. Linac‐integrated kV cone‐beam CT: technical features and first applications. Med Dosim. 2006;31(1):62–70. 16551530 10.1016/j.meddos.2005.12.008

[8] Mackie TR , Kapatoes J , Ruchala K , et al. Image guidance for precise conformal radiotherapy. Int J Radiat Oncol Biol Phys. 2003;56(1):89–105. 12694827 10.1016/s0360-3016(03)00090-7

[9] Pouliot J , Bani‐Hashemi A , Chen J , et al. Low‐dose megavoltage cone‐beam CT for radiation therapy. Int J Radiat Oncol Biol Phys. 2005;61(2):552–60. 15736320 10.1016/j.ijrobp.2004.10.011

[10] Sillanpaa J , Chang J , Mageras G , et al. Developments in megavoltage cone beam CT with an amorphous silicon EPID: reduction of exposure and synchronization with respiratory gating. Med Phys. 2005;32(3):819–29. 15839355 10.1118/1.1861522

[11] Langen KM , Meeks SL , Poole DO , et al. The use of megavoltage CT (MVCT) images for dose recomputations. Phys Med Biol. 2005;50(18):4259–76. 16148392 10.1088/0031-9155/50/18/002

[12] Yang Y , Schreibmann E , Li T , et al. Evaluation of on‐board kV cone beam CT (CBCT)‐based dose calculation. Phys Med Biol. 2007;52(3):685–705. 17228114 10.1088/0031-9155/52/3/011

[13] Lu W , Olivera GH , Chen Q , et al. Deformable registration of the planning image (kVCT) and the daily images (MVCT) for adaptive radiation therapy. Phys Med Biol. 2006;51(17):4357–74. 16912386 10.1088/0031-9155/51/17/015

[14] Chen J , Morin O , Aubin M , et al. Dose‐guided radiation therapy with megavoltage cone‐beam CT. Br J Radiol. 2006;79 Spec No1:S87–S98. 16980688 10.1259/bjr/60612178

[15] Stewart RD , Li XA . BGRT: Biologically guided radiation therapy – the future is fast approaching! Med Phys. 2007;34(10):3739–51. 17985619 10.1118/1.2779861

[16] Pouliot J . From Dose to Image to Dose: IGRT to DGRT. In: Mould RF, editor. Choices in advanced radiotherapy. Veenendaal (The Netherlands): Nucletron B.V.; 2007: 243–250.

[17] Morin O , Chen J , Aubin M et al. Dose calculation using megavoltage cone‐beam CT. Int J Radiat Oncol Biol Phys. 2007;67(4):1201–10. 17336221 10.1016/j.ijrobp.2006.10.048

[18] Spies L , Ebert M , Groh BA et al. Correction of scatter in megavoltage cone‐beam CT. Phys Med Biol. 2001;46(3):821–33. 11277228 10.1088/0031-9155/46/3/316

[19] Yoo S , Yin FF . Dosimetric feasibility of cone‐beam CT‐based treatment planning compared to CT‐based treatment planning. Int J Radiat Oncol Biol Phys. 2006;66(5):1553–61. 17056197 10.1016/j.ijrobp.2006.08.031

[20] van Zijtveld M , Dirkx M , Heijmen B . Correction of conebeam CT values using a planning CT for derivation of the “dose of the day”. Radiother Oncol. 2007;85(2):195–200. 17936387 10.1016/j.radonc.2007.08.010

[21] Aubry JF , Pouliot J , Beaulieu L . Correction of megavoltage cone‐beam CT images for dose calculation in the head and neck region. Med Phys. 2008;35(3):900–907. 18404926 10.1118/1.2839146

[22] Morin O . The Development and Clinical Role of Megavoltage Cone Beam Computed Tomography in Radiation Oncology. (PhD thesis, University of California, San Francsico, 2007).

[23] Siddon RL . Fast calculation of the exact radiological path for a three‐dimensional CT array. Med Phys. 1985;12(2):252–55. 4000088 10.1118/1.595715

[24] Perona P , Malik J . Scale space and edge detection using anisotropic diffusion. IEEE Transactions on Pattern Analysis and Machine Intelligence. 1990;12(7):629–39.

[25] Langen KM , Meeks SL , Poole DO , et al. The use of megavoltage CT (MVCT) images for dose recomputations. Phys Med Biol. 2005;50(18):4259–76. 16148392 10.1088/0031-9155/50/18/002

[26] Cho PS , Johnson RH , Griffin TW . Cone‐beam CT for radiotherapy applications. Phys Med Biol. 1995;40(11):1863–83. 8587937 10.1088/0031-9155/40/11/007

[27] Maltz J , Bose S , Shukla H et al. CT truncation artifact removal using water‐equivalent thicknesses derived from truncated projection data. Proceedings of the 29th IEEE Engineering in Medicine and Biology Society Conference. 2007 Aug 22–26; Lyon, France IEEE 2007 p. 2907–11. 10.1109/IEMBS.2007.4352937 18002603

[28] Petit SF , van Elmpt WJC , Nijsten SM , et al. Calibration of megavoltage cone‐beam CT for radiotherapy dose calculations: Correction of cupping artifacts and conversion of CT numbers to electron density. Med Phys. 2008;35(3):849–65. 18404922 10.1118/1.2836945

